# Shockwave generates < 100 > dislocation loops in bcc iron

**DOI:** 10.1038/s41467-018-07102-3

**Published:** 2018-11-16

**Authors:** Qing Peng, Fanjiang Meng, Yizhong Yang, Chenyang Lu, Huiqiu Deng, Lumin Wang, Suvranu De, Fei Gao

**Affiliations:** 1 Nuclear Engineering and Radiological Sciences, University of Michigan, Ann Arbor, MI 48109 USA; 2 School of Power and Mechanical Engineering, Wuhan University, 430072 Wuhan, China; 3 Shanghai Nuclear Engineering Research and Design Institute, 200233 Shanghai, China; 4 College of Materials Science and Engineering, Hunan University, 410082 Changsha, China; 5 Department of Mechanical, Aerospace and Nuclear Engineering, Rensselaer Polytechnic Institute, Troy, NY 12180 USA

## Abstract

The formation mechanism of < 100 > interstitial dislocation loops in ferritic steels stemming from irradiation remains elusive, as their formations are either too short for experiments, or too long for molecular dynamics simulations. Here, we report on the formation of both interstitial and vacancy dislocation loops in high energy displacement cascades using large-scale molecular dynamics simulations with up to 220 million atoms. Riding the supersonic shockwave generated in the cascade, self-interstitial atoms are punched out to form < 100 > dislocation loops in only a few picoseconds during one single cascade event, which is several orders of magnitude faster than any existing mechanisms. The energy analysis suggests that the formation of the interstitial loops depends on kinetic energy redistribution, where higher incidence energy or larger atom mass could improve the probability of the direct nucleation of interstitial dislocation loops.

## Abstract (web summary)

Irradiating iron introduces defects such as interstitial dislocation loops, whose exact formation mechanism remains unclear. Here, the authors use large scale molecular dynamics simulations to reveal a punch out mechanism that can directly create < 100 > interstitial dislocation loops.

## Introduction

Alloyed iron has incomparable hardness among all metals, in part because of the high density of dislocations^1–3^, a line defect in crystals, which can be formed by quenching, deformation, and irradiation^4^. Different types of dislocations in a crystal can be denoted by an atomic translation known as the Burgers vector. Dislocations can form 3D networks or 2D loops. Extensively consistent experiments have identified two kinds of dislocation loops, one with < 100 > and the other with 1/2 < 111 > Burgers vector, in body-centred cubic (bcc) iron using transmission electron microscopy (TEM) since 1960^5–11^. A dislocation loop can consist of interstitial atoms or vacancies^12^, whereas the prismatic interstitial type dislocation loops attract more attention due to their high mobility^1^. It is generally accepted that the formation energy of < 100 > loops is higher than < 111 > loops^13^, meaning that direct nucleation of < 100 > loops from interstitial atoms is highly unlikely. Although it has been suggested that the loops can be formed directly from bombardment^8^, there is no direct observation of the formations of < 100 > interstitial loops, neither from experiments nor molecular dynamics (MD) simulations in the past half century.

Five possible formation mechanisms have been proposed. We roughly denoted here as <110> shear, <111> reaction, <111> transformation, C15 transformation, and cascade overlapping, In 1965, Eyre and Bullough proposed that a <100> loop can form as a result of 1/2 < $1\overline 1 0$ > shear of a faulted 1/2 <110> loop^14^. Although this <110> shear mechanism obeys the Kirchinhoff law that conserves the burgers vectors, it has not been confirmed by MD simulations, as the proposed mechanism involves the formation of faulted loops, which is unfavourable due to high energy cost. Through atomistic modelling, Marian et al.^13^ proposed that two gliding <111> loops could meet and interact to form one <100> loop. Later, this loop formation was observed in Kinetic Monte Carlo simulations with stochastic components^15^. However, this <111> reaction mechanism needs stringent conditions, such as similar sizes and overlapping glide cylinders of the two colliding <111> loops. As a result, it is a rare event in reality^16^. Besides, the segments of <100> type dislocations formed in such a reaction mechanism are dynamically unstable and a single <111> dislocation loop eventually forms^17^. Chen et al. proposed a mechanism that <111> loops could transform from <111> loops directly at high temperatures^18^. They only justified it in a 4-SIA cluster, which seems to be challenging at larger sizes, since a junction has to form when the loop is partially reoriented, leading to a drastic increase in total loop length and energy. Recently, Zhang et al. proposed a mechanism that <100> loops can be transformed from a C15 Laves phase interstitial cluster^19,20^. This C15 transformation mechanism was further developed by ab initio calculations based discrete-continuum model^20^. Very recently, Granberg et al. proposed a mechanism of cascades overlapping with pre-existing radiation damage from MD simulations^21^. However, the dose rate is about nine orders of magnitude higher than that in any experiments. All these mechanisms assume thermodynamic equilibrium conditions and some precursors. Stimulations like stress^22^, temperature^9^, and impurities (carbon^17^, chromium^11^, helium^23^, etc) could change the relative stability of <100> over <111> clusters, and the rate of reaction or transformation, but not enough to explain their common occurrence of <100> loops under irradiation^17^. Nevertheless, all these proposed mechanisms require a long time (i.e. nanoseconds and above) due to the high energy barriers. In general, the energy barrier is more than tens of electronvolt for a nanometre size loop, making it infeasible to observe in the time scale of MD simulations^24^. It is worth noting that <100> interstitial loops have been observed by MD simulation of a single ion impact in bcc tungsten^25^. However, the atomic potential used for tungsten describes a <100> interstitial cluster to be stable than a <111> cluster, which is in contrast to ab initio calculations^26^. In addition, the kinetics are totally different because of the absence of subcascades in tungsten, as opposed to iron.

Here, we propose a punch mechanism for the formation of <100> loops by supersonic shockwave during high energy displacement cascade in bcc iron. This process occurs very quickly, in only a few picoseconds (ps, 10^−12^ seconds). In addition, the process does not require any precursors other than point defects. These two features make it distinguishable from all previous mechanisms. The supersonic shockwave generated in the cascade^27–29^ with 0.1 picoseconds is the stimulus, offering enough energy to nucleate the <100> loops directly from the pure lattices within high energy displacement cascades. Comparing to vacancy-type dislocation loops, the interstitial-type loops are more interesting because they are a feature of radiation damage and key media for material degradation, including irradiation-induced swelling, hardening, and plasticity^30^. Therefore, we focus on the self-interstitial type dislocations.

## Results

### Punch-out procedure

**Fig. 1:**
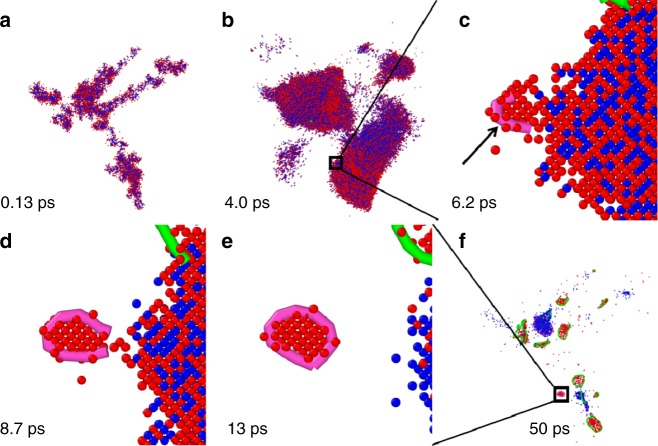
Punch-out procedure. A primary knocked-on atom with the kinetic energy of 200 keV at time zero moves along the <351> direction to initiate the cascade, with sequence of **a** subcascade, **b** thermal spike, **c** <100> interstitial dislocation loop nucleation, **d** formation, and **e** stabilization in the zoom-in region, and **f** the quenched stage. The snapshots are taken as side-views along the <010> direction of the cubic system, for better view of the <100> dislocation loops. Only the defects are displayed. The red particles stand for interstitial atoms and the blue particles are for vacancies. The black arrow is the eye-guide for the position of the nucleation of the <100> interstitial-type dislocation loops. The pink lines denote <100> dislocation loops and the green lines are for the <111> dislocation loops

We investigate the displacement cascades in pure bcc iron via molecular dynamics simulations at a temperature of 300 K. The high Primary Knock-on Atom (PKA) recoil energy E_PKA_ of 100, 150, and 200 keV are modelled within large simulation boxes containing 16,000,000 and 221,184,000 atoms. The high energy particle starts in the <351> direction from the left side of the simulation box at time zero. The nucleation process of a <100> interstitial loop in a displacement cascade with *E*_PKA_ = 200 keV is illustrated in snapshots in Fig. 1. For clarity, only part of the simulation cell is shown. The cascade procedure starts with supersonic shock wave at the speed of 831 km s^−1^. The initiation of the subcascades is illustrated in Fig. 1a, followed by the thermal spike shown in **b**. The < 100 > dislocation loop is generated right after the shock wave, as marked in **c**. The <100> loop is completely formed in **d**, at 13 ps after the collision and stabilized. There is no significant change in size and shape of the loop hereafter. The snapshot at time of 50 ps is in **f**. The whole movie is in Supplementary Movie 1. We extended our simulation time up to 1912 ps and still no much change occurs in this <100> interstitial loop.

We for the first time have directly observed the nucleation of the individual <100> interstitial loops in the displacement cascades in bcc iron. The nucleation starts right after thermal spike at 4.0 ps after the primary collision. Visible <100> dislocation segments are illustrated in **c-f** as the arrow pointed to. A complete individual <100> interstitial loop finally forms at 9 ps with the dislocation length of 4.4 nm. The nucleation is very fast, orders of magnitude faster than all previous proposed nucleation mechanism. This might be due to non-equilibrium process during the cascade, where high temperatures and high pressures could appear locally at the shockwave front. The dislocation length fluctuates around 4.4 nm. There is no obvious growth within our simulation time. Once the individual <100> interstitial loop nucleates, it is much stable compared to point defects and <111> dislocation loops.

### Punch-out mechanism

**Fig. 2:**
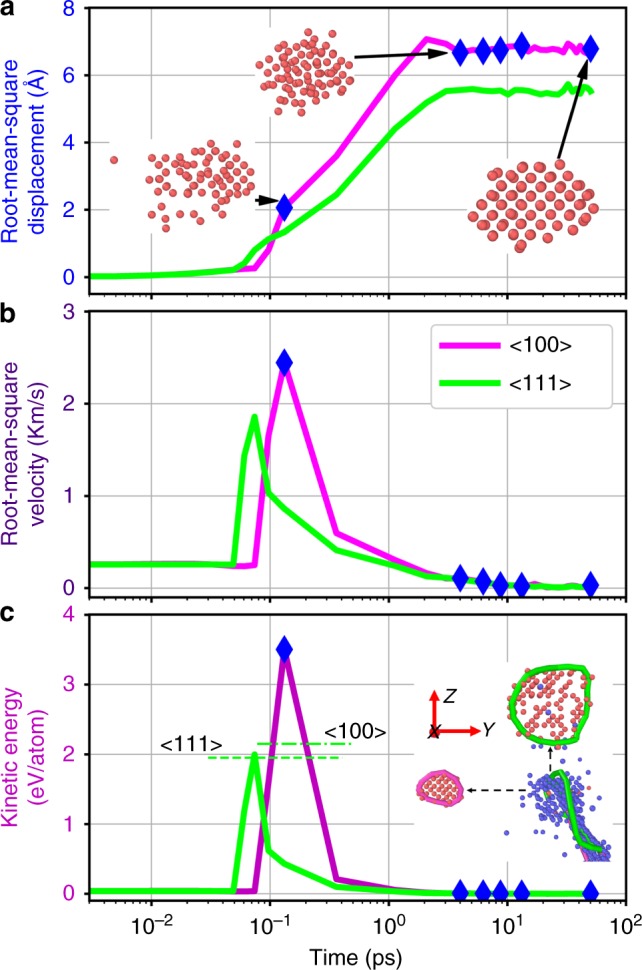
Punch-out mechanism. One <100> (red) and one <111> (green) interstitial loop are selected for analysis of their **a** the root-mean-square displacement, **b** root-mean-square velocity, and **c** averaged kinetic energy per atom as a function of time for the interstitial atoms forming the dislocation loops. The insets in **a** show the morphology of the SIA atoms consisting of the <100> loop at three different stages. The blue diamond dots mark the corresponding locations in the curves for the snapshots in Fig. 1. The dash-lines in **c** mark the formation energies of the dislocation loops per atom. The selected <100> and <111> interstitial loops are in the inset of **c** with coordination orientation. The red dots denote the interstitial atoms and the blue dots denote the vacancies. The <100> loop and <111> loop are represented by the red and green lines, respectively

In order to gain more insight into the formation mechanism, we examine two individual loops, one <100> (red) and one <111> (green) loop, shown in the bottom right corner of Fig. 1f. They are selected intentionally because they nucleate almost simultaneously in the same subcascade. The same formation environment makes them ideal objects to study the formation mechanism. The zoom-in figure with orientation mark is displayed in the inset of Fig. 2c. The number of interstitial atoms contained in the loop is 40 and 108 for <100> and <111> loop, respectively, with loop perimeter 4.3 and 9.4 nm, and diameter of 1.4 and 3.0 nm, respectively. We trace the positions of these interstitial atoms with the advantage of MD simulations. The root-mean-square displacement (RMSD), referring to the position at time 0 (right before the collision), is displayed in Fig. 2a as a function of time for both the <100> loop and the <111> loop. The large portion of displacement (>95%) completes in a very short time, about 2 ps for both loops. After that, the interstitial atoms only process local rearrangement, without much change in their positions. The snapshots of the interstitial atoms composing the <100> loop are projected to a (110) plane as the insets. These interstitial atoms are punched out and ejected within a couple of picoseconds. Then the < 100 > loop formed after local relaxation without much change of their positions. The <111> loop starts moving earlier, but the final RMSD is less than that of the <100> loop. It is also reflected in the inset of Fig. 2c, where the <111> loop (green) is closer to the collision centre (blue) than the <100> loop.

The shockwave strength is associated with the atoms’ velocity. The root-mean-square velocity (RMSV) is a measure of the average velocity of an object, which is obtained directly from time derivative of the root-mean-square displacement. The nucleation of the <111> interstitial loop initiates at 0.049 ps. With the supersonic wave generated from the collision, all the 108 interstitial atoms gain the RMSV as high as 3.72 km s^−1^ at 0.074 ps. The nucleation of the <100> loop starts a little bit later at 0.09 ps, but the 40 interstitial atoms forming the <100> loop gain a higher RMSV of 4.89 km s^−1^ at 0.132 ps. This high RMSV reflects the dislocation formation mechanism that is punched out by shockwaves. After the punch, the RMSV quickly reduces to 0.1 km s^−1^, indicating the loops experience thermal vibrations thereafter, consistent with the picture of their positions. The dynamic procedure of the shockwave generating dislocation loops is also illustrated in Supplementary Movie 2.

It is worth noting that the mechanism for dislocation generation in shockwave deformation has been proposed 40 years ago^31^. It is well accepted that shock compression introduces homogeneous nucleation of dislocations in crystals^32^. Here the shockwaves generated in the displacement cascade are localized, heterogeneous, and complicated because of the subcascades: multiple shockwaves are generated during the process at the time scale of picoseconds and length scale of nanometres.

Both the <100> and the <111> loops nucleate in the same collision, sharing the same depleted zone (blue region in the inset of Fig. 2c). One might be curious about the fundamental rule that determines the types of these shockwave induced dislocation loops. To that end, we examine the energies of the selected <100> and <111> interstitial loops. The kinetic energies of interstitial atoms in the loop are summed up at each time step during the cascade. The average kinetic energies are elucidated in Fig. 2c for both the <100> and the <111> loops. The maximum mean kinetic energy of the <100> loop is 3.5 eV per atom at the time of 0.132 ps, and the counterpart of the <111> loop is 2.0 eV per atom at the time of 0.074 ps. During the cascade, the nucleation of interstitial dislocation loops includes two parts: the generation of interstitials (vacancies) first and then formation of the loop. Therefore, the formation energy of the interstitial dislocation loops from a pure crystal system should also include the formation of vacancies and the interstitials. The vacancy formation energy is 1.718 eV, agreeing with the experimental value measured by positron annihilation^33^. With a scaling value of the formation energy of interstitial loops^20^, the total formation energy is 4.14 eV and 3.94 eV per atom for the <100> loop and the <111> loop, respectively (Fig. 2c). It is evident that the punch provides enough kinetic energy to form the <100> loop within high energy cascade. On the contrary, the average kinetic energy gained during the punch is not sufficient for the nucleation of a <100> loop, resulting the nucleation of the <111> loop instead (Fig. 2c).

It is worth pointing out that the average kinetic energy is more than that needed to form the <100> loop as shown in Fig. 2c. One might expect that the nucleation of the <100> interstitial loop could occur in a lower PKA recoil energy. We later confirm that one individual <100> interstitial loop nucleates in *E*_PKA = _150 keV cascades. However, we have not observed the nucleation of individual <100> interstitial loops for all 10 cascades simulations with the PKA recoil energy of 100 keV. It is well known that the morphology and the distribution of the debris are very complex^27–29^ due to the subcascade formation and the occurrence of channelling, in addition to the anisotropic nature of crystal structure. As a result, a deterministic redistribution of the injected PKA energy is impossible. The cascade procedure differs from case to case in stochastic nature. In fact, we have done 10 cases of displacement cascades of the same PKA recoil energy and temperature but on different PKA atoms and directions (all along <351> directions). Only 7 out of 10 cases have the individual <100> interstitial dislocation loops nucleated. As a comparison, it is 1 and 0 out of 10 cases for *E*_PKA_ = 150 and 100 keV, respectively. Coincidentally, the energy cutoff in observation of <100> interstitial loops in this study agrees with the experiments, as the first report of the <100> interstitial loops in iron also utilized 150 keV Fe^+^ ion irradiation^6^.

Obviously, increase of the PKA recoil energy can augment the probability of the nucleation of <100> interstitial loops. Since the kinetic energy provides the main source of generating <100> interstitial loops, the increase of the efficiency of kinetic energy transfer during the collision is another way to enlarge the nucleation probability of <100> interstitial loops. This could be achieved by the heavy ion bombardment means surface interaction. Actually, by artificially increasing the PKA atom mass, a recoil energy as low as 20 keV have been reported to generate <100> interstitial loop in a MD study^34^. The reason is that the large mass makes the velocity smaller for the same kinetic energy, leading to smaller damage volume and high kinetic energy density, which in turn boosts the nucleation of the <100> interstitial loops. In other words, the efficiency and population of the <100> loops could be tuned by the PKA recoil energy and the incident ion mass or species.

One might be concerned with whether the formation of <100> interstitial loops described above is an artefact of the interatomic potential used. To check that, we repeat the displacement cascade simulations using M07^35^, M11^2^, and Ackland04^36^ potentials. In all potentials, the <100> interstitial loops were observed in cascade simulations with *E*_PKA_ = 200 keV. Therefore, the punch-out mechanism for the nucleation of <100> interstitial loops in high energy displacement cascades of 200 keV in iron is not limited to the Mendelev potential we used.

### After punch-out

**Fig. 3:**
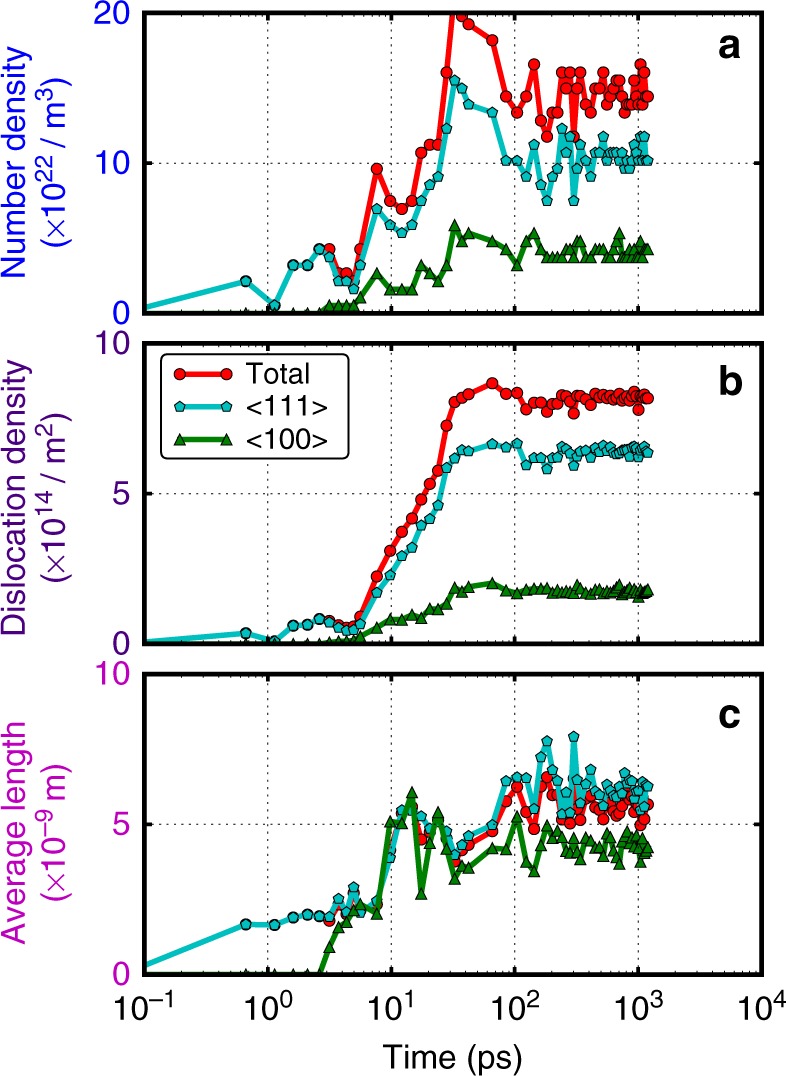
Dislocation density. **a** The number density; **b** Density; **c** Average length of the dislocations of <100> and <111> types and total as a function of time

Besides the nucleation, the growth and evolution of microstructures are important in governing the materials’ properties. There are about 56 dislocation loops nucleated in the cascade shown in Fig. 1, including both vacancy loops and interstitial loops, <111> and <100> types, full loops and segments. The dislocation number density (number of dislocations per volume), dislocation density (total dislocation length per volume) and the average length of dislocations are displayed in the panel of Fig. 3a–c respectively, as a function of time. The number densities have a much larger fluctuation before 100 ps and reach a plateau thereafter. The number density ratio of <111> to <100> loops is about 2.5:1. The <111> dislocation loops are dominant with about three times more than the <100> loops. The individual dislocation loop might change length and positions after nucleation. However, the dislocation densities are quite conservative in our MD simulation time up to 1200 ps. Referring to the number density curves, the dislocation density ones are much smoother, and they are stable after 100 ps. The dislocation density of <111> loops takes 78.3% of the total, meaning that the ratio of <111> to <100> loops is about 4:1. The average length of the dislocation loops is 5.6, 6.3, and 4.3 nm for total, <111> and <100> loops, respectively, in this case. The average length of <100> loop in our studies is smaller than that of <111> loops and the diameter is 1.4 nm.

In addition to the fundamental understanding of displacement cascade that we discussed above, this proposed punch-out mechanism could have interesting and promising applications. The sessile <100> loops are immobile compared to the <111> loops due to the much higher formation energy and migration barrier. As a result, it can serve as an additional sink to point defects and enhance the recombination with the vacancies, while the <111> loops can be quickly absorbed by grain boundaries and dislocations due to their small migration energies. Therefore, more vacancies will be eliminated during the recombination process, effectively reducing the swelling. For the same reason, with the enhanced recombination of interstitials and vacancies, the embrittlement will be reduced with less defects. Therefore, it might be a feasible avenue to fabricate advanced steels with high resistance to both swelling and hardening by engineering the <100> dislocation loops via high energy particle bombardment, heavy-ion implantation^37^, laser shock, and other shock loadings.

## Discussion

In summary, through large-scale molecular dynamics simulations of high energy displacement cascades, we have observed the nucleation of <100> interstitial dislocation loops. Based on the velocity and energy analysis, we propose the punch-out mechanism of the formation of <100> interstitial dislocation loops through supersonic shockwaves, which is directly nucleated within a few picoseconds during displacement cascade. The new mechanism provides more insight in engineering micro-structures via ion implantation, high energy particle bombardment, or shockwave compression.

## Methods

### Molecular dynamics simulations

The classical molecular dynamics simulations are carried out using the Large-scale Atomic/Molecular Massively Parallel Simulator (LAMMPS)^38^, accompanying with the embedded-atom-method (EAM) empirical potential^39,40^ for atomic interactions. We choose this potential because it provides correct description of the relative stability of SIAs of different configurations and the vacancy migration energy. The universal screened-Coulomb potential was employed to describe the short-range (<0.1 nm) interatomic interaction in high-energy atomic collisions in cascades. In the middle range (from 0.10 to 0.20 nm), the interatomic interaction is a hybrid of the two potentials by an exponential spline.

Large simulation boxes of 200*a*_0_ × 200*a*_0_ × 200*a*_0_, 384*a*_0_ × 384*a*_0_ × 384*a*_0_, and 480*a*_0_ × 480*a*_0_ × 480*a*_0_ in bcc structure containing 16,000,000,113,246,208, and 221,184,000 iron atoms, respectively, are used for the classical molecular dynamics simulations of the high energy displacement cascade at temperature of 300 K. *a*_0_ is the lattice constant with the value of 0.287 nm. The side length of the cubic MD simulation box is 57.3, 110.2, and 137.8 nm, respectively. The size effect was examined comparing to a larger simulation box of 384*a*_0_ × 384*a*_0_ × 384*a*_0_ with 113,246,208 atoms and the side length is 110 nm. The PKA recoil energies of 100, 150, and 200 keV are examined.

Prior to initiating the cascade, a block was equilibrated for 40 ps at 300 K. This initial atom block was then used as a starting point for the cascade simulation and a reference for the defect analysis. The cascade was initiated by imparting a kinetic energy E_PKA_ to the selected primary knock-on atom (PKA) along a high-index direction. To avoid channelling by the PKA, a high-index direction <135> was used. Integration of the equations of motion of atoms was performed using a leapfrog algorithm with variable time-step, determined by limiting the displacement of the fastest atom to 0.0166*a*_0_ in one time-step. This number is carefully selected as the long time (40 ps) average of the maximum displacement of simulation system with 16,000,000 iron atoms after thermodynamical equilibrium before the cascade simulations.

No electronic stopping or electron-phonon coupling was included in the simulations. Zarkadoula et al. employed a two-temperature model to study the effect of electronic stopping on defect production in bcc iron^29,41,42^, and reported that there is no substantial difference in defect generation with and without electronic stopping for the recoil energies ranged from 200 to 500 keV^29^. In addition, it has been noted that the electron-phonon coupling has no substantial effect on the final damage for examined recoil energies of 100 and 200 keV in bcc iron^41^. Furthermore, there is a parameter, known as cutoff kinetic energy, for electronic stopping in MD modelling. A value of 5.4 km s^−1^ or higher is generally adopted for iron^41,42^, which is higher than the shock speed of 4.89 km s^−1^ for a <100> loop formation. Therefore, considering the electronic energy loss will not significantly change our main conclusions.

All results were obtained in the micro-canonical ensemble (NVE), with periodic boundary conditions. The NVE boundary conditions are well examined. The boost of the instantaneous temperature and pressure is 13.9 K and 0.048 GPa, respectively, at the thermal spike stage, which is negligible. The variation of temperature and pressure of the system as a function of time during a displacement cascade are illustrated in Supplementary Figure 1 and Supplementary Figure 2, respectively. The long-range elastic strain has negligible effect on the generation of the <100> interstitial loops in our model. The Wigner-Seitz defects were calculated using Voronoi cell analysis^43^ in OVITO^44^. The dislocation analysis were performed using OVITO^45^. These settings have successfully employed in previous displacement cascade studies.

## Electronic supplementary material

Supplementary Information

Description of Additional Supplementary Files

Supplementary Movie 1

Supplementary Movie 2

## Data Availability

The data that support the findings of this study are available in Mendeley Data with the identifier 10.17632/pk5kkvtdb5.1
