# Supplementary material for: Shockwave generates < 100 > dislocation loops in bcc iron
Source: Nat Commun. 2018 Nov 16;9:4880. doi: 10.1038/s41467-018-07102-3 (PMC6240105; doi:10.1038/s41467-018-07102-3)
Supplement: Supplementary file 1 — Supplementary Information [file 41467_2018_7102_MOESM1_ESM.pdf]

Peng, *et al.* Shockwave Generates <100> Dislocation Loops in bcc Iron

Supplementary Figures

Supplementary Figure 1: Instantaneous temperature during cascades. All molecular dynamics (MD) simulations of the displacement cascade in this study are performed in NVE micro-canonical ensemble, with periodic boundary conditions. The cascade temperature is 300K. We found the temperature change at the thermal spike stage is 14K, as shown in Figure S1. The system temperature quickly drops to 305.5K after thermal quench stage, followed by slowly decrease with fluctuations during relaxation stage. The irradiation experimental precision in temperature control is around 10K in general. It is very common that the temperature rise up a few hundred degrees, for example in Ref [1]. Therefore, the effect of temperature on our boundary condition is negligible.

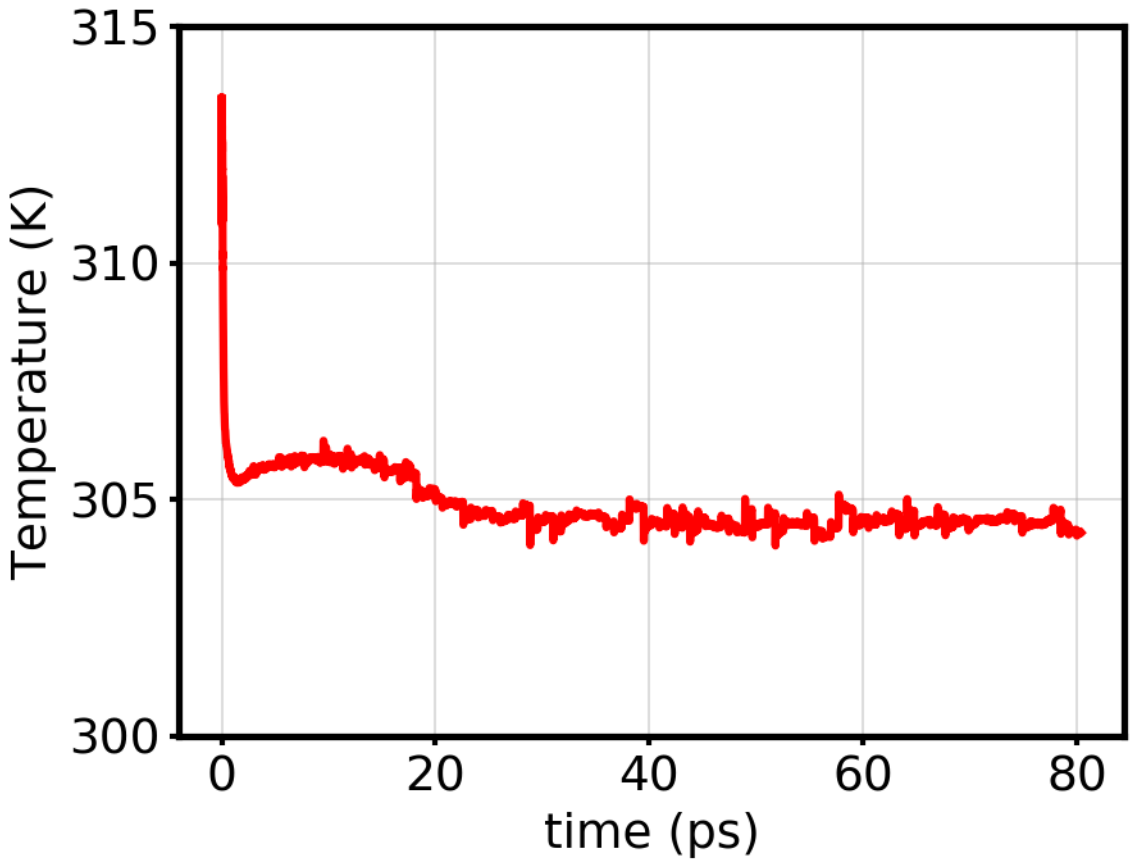

Supplementary Figure 1: The system temperature as a function of time during a displacement cascade with the primary recoil energy of 200 KeV.

Supplementary Figure 2. Instantaneous pressure during cascades

We also examined the change of pressure as a function of time during the displacement cascade. The pressure change is within 0.05 GPa at the thermal spike stage and quickly decay to 0.03 GPa after thermal quench. Considering the precision of pressure control in a typical NPT ensemble is 0.1 GPa, the 0.05 GPa pressure pulse is negligible comparing to thermal vibrations.

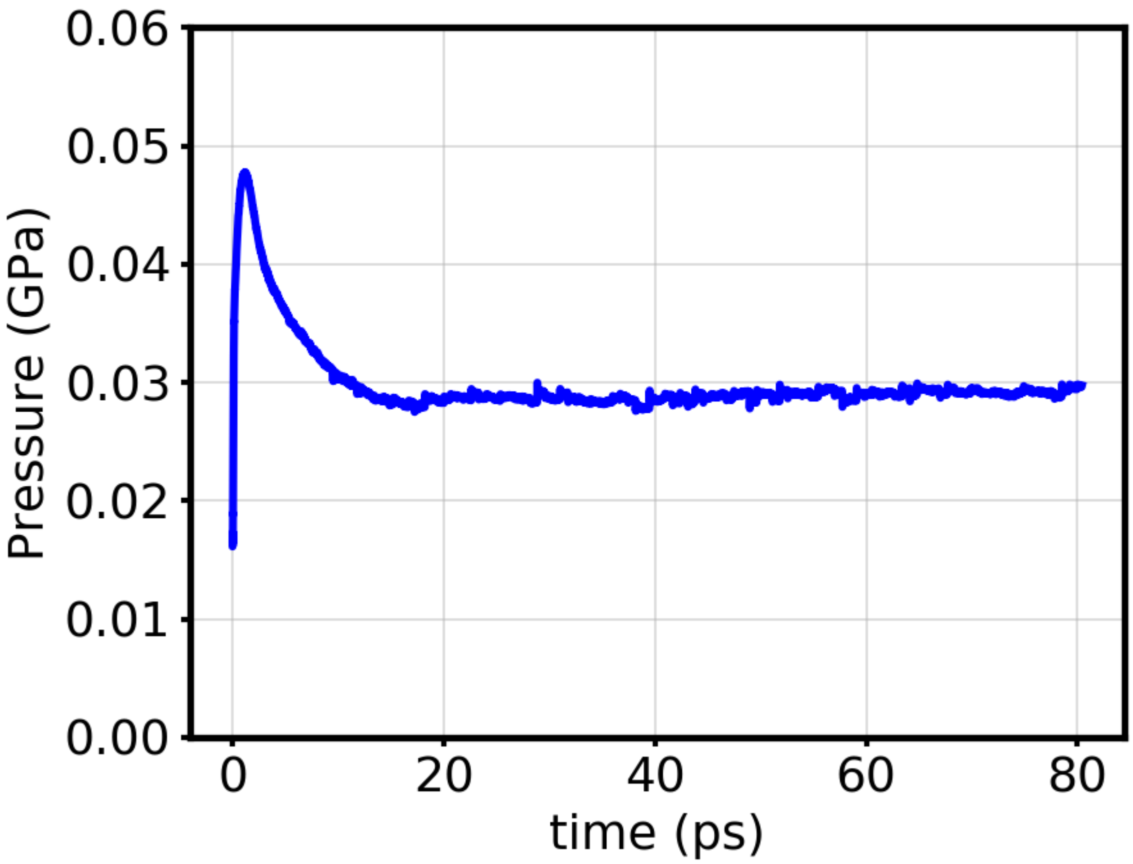

Supplementary Figure 2: Instantaneous pressure during cascades. The system pressure as a function of time during a displacement cascade with the primary recoil energy of 200KeV.

Supplementary References

1. Granberg, F., Byggmastar, J., Sand, A. E. & Nordlund, K. Cascade debris overlap mechanism of <100> dislocation loop formation in Fe and FeCr. *EPL* **119,** 56003 (2017).
