# Supplementary material for: Shockwave generates < 100 > dislocation loops in bcc iron
Source: Nat Commun. 2018 Nov 16;9:4880. doi: 10.1038/s41467-018-07102-3 (PMC6240105; doi:10.1038/s41467-018-07102-3)
Supplement: Supplementary file 2 — Description of Additional Supplementary Files [file 41467_2018_7102_MOESM2_ESM.docx]

**Description of Additional Supplementary Files**

File Name: Supplementary Movie 1

Description: **Cascade procedure.** A displacement cascade in pure bcc iron is illustrated via molecular dynamics simulations at temperature of 300 K, where only defects are displayed. The recoil energy of Primary Knock-on Atom (PKA), EPKA, is 200 keV. The PKA starts in the direction from left side of the simulation box at time zero, where only part of the simulation cell is shown for clarity. The cascade procedure starts with supersonic shock wave at the speed of 831 km s-1 . The whole cascade procedure is displayed up to 50.2 ps, showing the fast generation of the interstitial loops via a punch-out mechanism. The red dots stand for interstitial atoms and the blue dots are for vacancies. The pink lines denote dislocation loops and the green lines are for the dislocation loops. The red arrows mark the Burgers vectors of the dislocations.

File Name: Supplementary Movie 2

Description: **The shockwaves in a cascade.** The shock waves during the displacement cascade of EPKA = 200 keV at 300 K are displayed by the high velocity atoms (red spheres). The pink lines denote dislocation loops and the green lines are for the dislocation loops. The red arrows mark the Burgers vectors of the dislocations.
